# Protein quality assessment with a loss function designed for high-quality decoys

**DOI:** 10.3389/fbinf.2023.1198218

**Published:** 2023-10-17

**Authors:** Soumyadip Roy, Asa Ben-Hur

**Affiliations:** Department of Computer Science, Colorado State University, Fort Collins, CO, United States

**Keywords:** protein structure quality assessment, deep learning, graph convolutional networks, epsilon-insensitive loss function, critical assessment of structure prediction

## Abstract

**Motivation:** The prediction of a protein 3D structure is essential for understanding protein function, drug discovery, and disease mechanisms; with the advent of methods like AlphaFold that are capable of producing very high-quality decoys, ensuring the quality of those decoys can provide further confidence in the accuracy of their predictions.

**Results:** In this work, we describe Q_*ϵ*_, a graph convolutional network (GCN) that utilizes a minimal set of atom and residue features as inputs to predict the global distance test total score (GDTTS) and local distance difference test (lDDT) score of a decoy. To improve the model’s performance, we introduce a novel loss function based on the *ϵ*-insensitive loss function used for SVM regression. This loss function is specifically designed for evaluating the characteristics of the quality assessment problem and provides predictions with improved accuracy over standard loss functions used for this task. Despite using only a minimal set of features, it matches the performance of recent state-of-the-art methods like DeepUMQA.

**Availability:** The code for Q_*ϵ*_ is available at https://github.com/soumyadip1997/qepsilon.

## 1 Introduction

Predicting a protein’s 3D structure from its amino acid sequence has been an area of avid interest for many years (Al-Lazikani et al., 2001). Recently, significant progress has been made in this field with the introduction of AlphaFold, a deep learning system that achieved remarkable accuracy in predicting protein structures (Jumper et al., 2021). While experimental identification of native protein structures remains a time-consuming and costly process, computational methods have made it possible to generate thousands of tertiary structures, known as decoys, in a matter of hours (Shehu, 2015). However, identifying the best structure remains a challenge. Therefore, it is necessary to employ a quality assessment stage to identify high-quality, near-native decoys among the generated decoys (Akhter et al., 2020). This remains true even with AlphaFold’s recent breakthrough performance (Chen et al., 2023). Furthermore, with the subsequent availability of genome-wide predicted structures across many species (Varadi et al., 2022), the quality assessment problem is as relevant as ever.

In this work, we address the decoy quality assessment problem with the help of graph convolutional networks (GCNs); we introduce a novel loss function inspired by the support vector regression, *ϵ*-insensitive loss function, that is designed to take into account our intuition about what makes a good quality assessment predictor, namely, that it focuses on making correct predictions for those decoys that matter: decoys with high quality. We compare our method, called Q_*ϵ*_, to other state-of-the-art methods and demonstrate that our method outperforms most of those methods while using only a very basic set of features computed from a decoy’s sequence, without the need for engineered features.

## 2 Related work

Current techniques for quality assessment can be divided into two categories. One is single-model methods that operate on single structural models to estimate their quality (Wallner and Elofsson, 2003). The second category consists of methods that use consistency among several candidates to estimate quality (Lundström et al., 2001). Protein quality assessment methods have been evaluated in the Critical Assessment of Structure Prediction (CASP) competition (Moult et al., 1995) since CASP7. The CASP13 single-model methods, the focus of this work, performed comparably or better than consensus methods for the first time (Cheng et al., 2019). A variety of single-model approaches have been proposed, and currently, machine learning-based methods dominate this area.

Until a few years ago, methods that use standard machine learning techniques with a large collection of engineered features computed from sequence and structure were the prevalent approaches for quality assessment. The ProQ series of methods (ProQ, ProQ2, ProQ3, and ProQ3D) (Uziela et al., 2017) used features such as the distribution of atom–atom contacts, residue–residue contacts, solvent accessibility, secondary structure, surface area, and evolutionary information. ProQ3 (Uziela et al., 2016) also incorporated features based on Rosetta energies. ProQ3D (Uziela et al., 2017) used the descriptors of ProQ3 as inputs in conjunction with a multi-layer perceptron and was one of the top performers of CASP13.

The current state-of-the-art method for quality assessment uses deep learning, including various types of 3D convolutional networks and graph neural networks, which have been demonstrated to be effective tools for modeling protein 3D structures (Derevyanko et al., 2018; Fout et al., 2017). Deep convolutional networks as a tool for the representation of decoy structures were introduced by Derevyanko et al. (2018). Their method, 3DCNN, used 3D convolutional networks applied to a volumetric representation of a decoy structure. The Ornate method by Pagès et al. (2019) improved upon 3DCNN by defining a canonical orientation for each residue. The GraphQA method by Baldassarre et al. (2020) employed a graph convolutional network with an extensive number of engineered features and achieved state-of-the-art performance on CASP13 decoys. Chen et al. (2023) used a graph neural network to estimate the accuracy of AlphaFold models, which is one of the current state-of-the-art methods, and improved on the results obtained with DeepAccNet by Hiranuma et al. (2021) while borrowing many ideas from its architecture. They used a combination of categorical loss and L2-loss on the lDDT scores to distinguish between decoys of varying quality levels. The DeepUMQA method uses 3D convolution over a collection of residue-level engineered features (Guo et al., 2022), and its successor, DeepUMQA2 (Liu et al., 2023), is also a state-of-the-art performer.

Most existing methods for quality assessment rely on engineered features. In contrast, our approach uses sequence embeddings computed using protein language models; convolutional layers applied to both atomic- and residue-level graphs are then used to put them in the context of the decoy structure. In combination with a novel loss function specifically designed for the quality assessment problem, our method can outperform the recent DeepUMQA method (Guo et al., 2022).

## 3 Methods

### 3.1 The quality assessment problem

Computational methods for predicting a protein’s 3D structure produce large numbers of decoy conformations for a given target protein. In quality assessment, we seek to rank these decoys based on their similarity to the experimentally determined native structure. We address this as a regression problem: our method is designed to predict the global distance test total score (GDTTS) (Zemla, 2003) and the local distance difference test (lDDT) score (Mariani et al., 2013), which are the official CASP scores for global-level decoy quality. While several recent methods were designed to predict the lDDT score (Hiranuma et al., 2021; Chen et al., 2023), we used both scores to allow for direct comparison with GraphQA, which is the most similar approach to the method presented here and would allow us to compare with more recent QA methods like DeepUMQA and DeepUMQA2. GDTTS measures the percentage of residues in the superimposed predicted structure that are within a certain distance threshold of their corresponding residues in the true structure. lDDT score is a superposition-free score that represents the local distance difference among all atoms in a predicted structure, thereby providing an idea of the local quality of the predicted structure. Decoy structures with high GDTTS and lDDT score ( > 0.85) indicate that they closely resemble the native structure. In what follows, we describe Q_*ϵ*_, a graph convolutional network that is trained on labeled decoy 3D structures, utilizing a basic set of features generated from atoms and residues using a combination of the L1-loss and a modification of the SVM regression *ϵ*-insensitive loss function (Drucker et al., 1996).

### 3.2 Atom- and residue-level graph convolution

**FIGURE 1:**
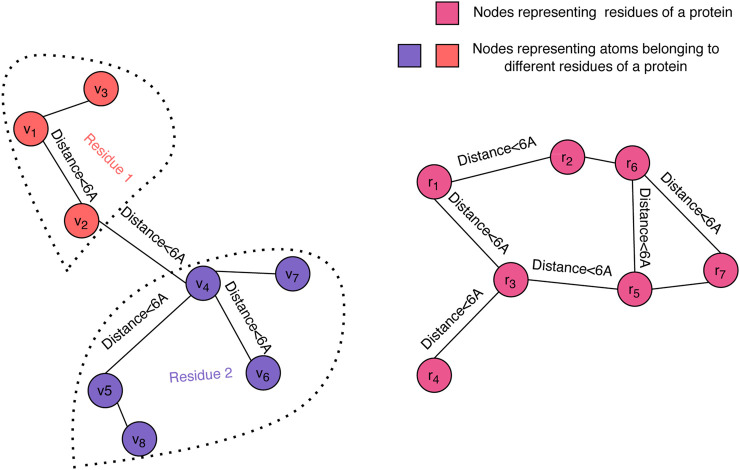
Graph representation of a decoy structure. The structure of a decoy is represented using two graphs: one at the atomic level (left) and one at the residue level (right). Our graph convolution operation at the atom level differentiates between edges within a residue and edges across neighboring residues.

Graph convolution is a powerful approach for representing protein 3D structures (Fout et al., 2017) and has proven its value for the quality assessment problem (Baldassarre et al., 2020). In order to enable us to forgo engineered features, we have chosen to represent the 3D structure of a decoy using dual graphs at the atom and residue levels (see Figure 1). Each of the graphs is a nearest neighbor graph where a pair of nodes is connected by an edge if their distance in the structure is less than a given threshold, where 6Å was the selected value in our experiments, and the distance between residues is the minimum distance between their atoms. We used up to 20 nearest neighbors to define the edges in both the atom-level and the residue-level graphs.

We perform graph convolution separately at the atom and residue levels. First, we describe the atom-level graph convolution (GCN_atom_). Each atom *i* is assigned a feature vector $v_{i}^{\left(l\right)}$ that contains the features for layer *l* of graph convolution. The representation of a source atom $v_{i}^{\left(l\right)}$ is updated based on its neighbors within the same residue $\left(N^{\left(s\right)}\left(i\right)\right)$ and the neighbors across residues $\left(N^{\left(o\right)}\left(i\right)\right)$ according to

$$v_{i}^{\left(l+1\right)}=ReLU(W_{l}^{\left(c\right)}v_{i}^{\left(l\right)}+\frac{1}{\left|N^{\left(s\right)}\left(i\right)\right|}W_{l}^{\left(s\right)}\underset{j\in N^{\left(s\right)}\left(i\right)}{\sum }v_{j}^{\left(l\right)}+\frac{1}{\left|N^{\left(o\right)}\left(i\right)\right|}W_{l}^{\left(o\right)}\underset{j\in N^{\left(o\right)}\left(i\right)}{\sum }v_{j}^{\left(l\right)}+b_{v}^{\left(l\right)}),$$ (1)

where $W_{l}^{\left(c\right)}$ is the weight matrix with respect to the source atom in layer *l*, $W_{l}^{\left(s\right)}$ is the weight matrix with respect to the neighboring atoms in layer *l* within the same residue as that of the source atom, $W_{l}^{\left(o\right)}$ is the weight matrix with respect to the neighboring atoms in layer *l* that belong to a different residue than the source atom, and finally, $b_{v}^{\left(l\right)}$ is the bias in layer *l* for the atom-level GCN. The inputs to the atom-level convolution are derived from one-hot encoding of the atom type as described in the following sections.

In parallel to the atom-level convolution, we perform convolution over the residues that make up a decoy structure. This operation, denoted as GCN_residue_, is used to update the residue-level representation $r_{i}^{\left(l\right)}$ , which is the feature vector for residue *i* in layer *l* of the network. This operation is defined as follows:

$$r_{i}^{\left(l+1\right)}=ReLU\left(W_{l}^{\left(cr\right)}r_{i}^{\left(l\right)}+\frac{1}{\left|R\left(i\right)\right|}W_{l}^{\left(r\right)}\underset{j\in R\left(i\right)}{\sum }r_{j}^{\left(l\right)}+b_{r}^{\left(l\right)}\right),$$ (2)

where R(i) is the set of the neighboring residues of residue *i*, $W_{l}^{\left(cr\right)}$ is the weight matrix with respect to the source residue in layer *l*, $W_{l}^{\left(r\right)}$ is the weight matrix with respect to the neighboring residues in layer *l*, and $b_{r}^{\left(l\right)}$ is the bias in layer *l*. The inputs to the residue-level convolution are embeddings computed using ProtTrans (Elnaggar et al., 2021) as described in the following sections.

### 3.3 Network architecture

**FIGURE 2:**
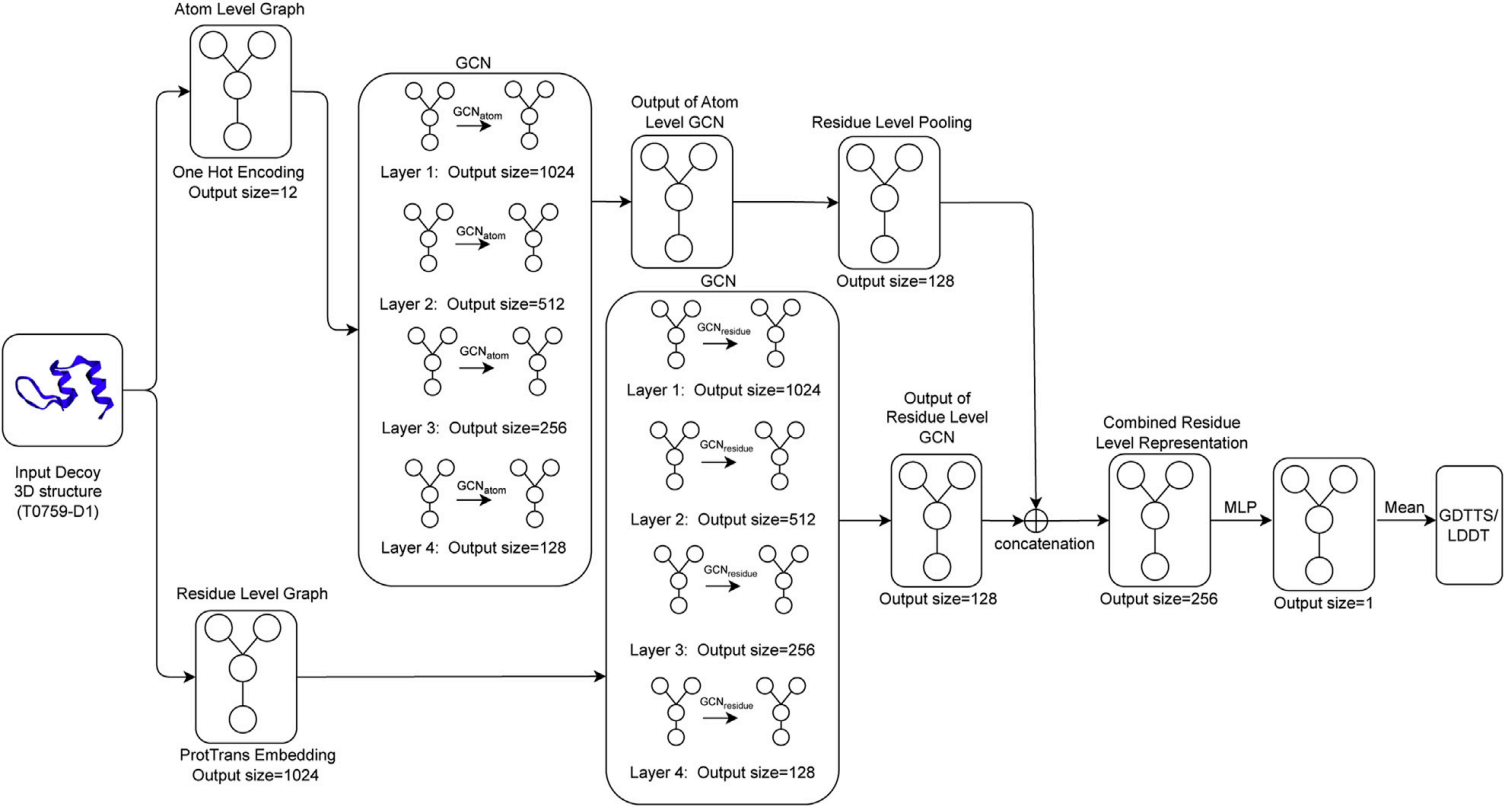
*Q*_*ϵ*_ model architecture illustrating how an input decoy structure is propagated through multiple graph convolutional layers (*GCN*_*atom*_ for the atom-level representation and *GCN*_*residue*_ for the residue-level representation of a protein); the outputs of the two sets of convolutional layers are concatenated and fed through a multi-layer perceptron (MLP) to generate local scores that are then averaged to compute the predicted GDTTS or lDDT score.

The architecture for *Q*_*ϵ*_ includes four graph convolutional layers that aggregate information at the atomic level (*GCN*_*atom*_) and four graph convolutional layers that pass information at the residue level (*GCN*_*residue*_). To ensure model stability and generalization, we apply batch normalization (Ioffe and Szegedy, 2015) after each application of an activation function to normalize the activations across the nodes in the graph. To create a coherent representation at the residue level, we apply a maximum pooling operation to the output of the final layer of *GCN*_*atom*_. The final residue-level representation is obtained by concatenating the output of the pooled atomic-level convolution and the output from the residue-level GCN. This concatenated output is passed through a multi-layer perceptron (MLP), which outputs a single output per residue of the decoy structure. The final output of the network, which is our predicted value of GDTTS or lDDT score, is then produced by averaging over the node-level scores. This process is shown in Figure 2.

### 3.4 Atom and residue features

Our method performs convolution at both the atom and residue levels. Here, we describe the features used at both levels.

#### 3.4.1 Atom features

We represent the atoms using one-hot encoding by grouping atoms into 11 different types (Derevyanko et al., 2018). This grouping reflects both the type of atom (carbon, oxygen, or nitrogen) and its context within the residue (e.g., alpha carbon or the different group an atom belongs to). In doing so, we are able to incorporate important information of the atoms while also capturing the relationships between the atoms and their corresponding residues.

#### 3.4.2 Residue features

We compute residue features by feeding the amino acid sequence of a decoy to the ProtTrans protein language model (Elnaggar et al., 2021). ProtTrans embeddings provide a very useful representation of the amino acid sequence, capturing relationships between residues and their structural context (Elnaggar et al., 2021). We take the embeddings from the last hidden state of the transformer attention stack of the ProtTrans model, with an output embedding of 1,024 dimensions, which serves as the input to the residue-level GCN.

### 3.5 A modified *ϵ*-insensitive loss

**FIGURE 3:**
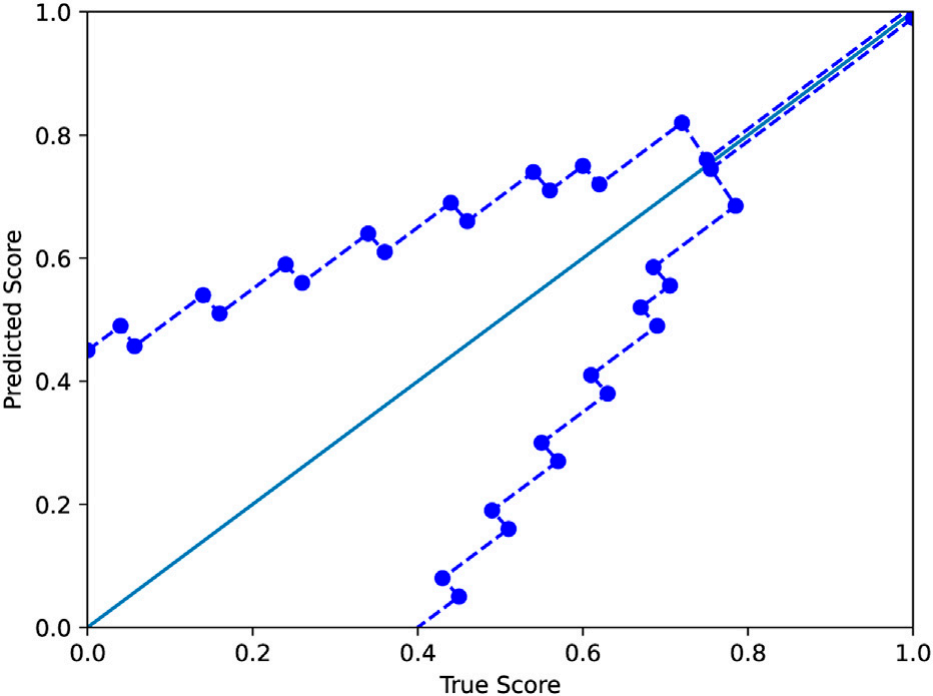
The modified *ϵ*-insensitive loss uses a variable-sized band around the diagonal in which a predicted score is not penalized. The band becomes smaller as the GDTTS or lDDT score increases, reflecting our expectation for precise predictions for decoys that are closer to the native structure.

In this work, we address quality assessment as a regression problem with the objective of predicting GDTTS or lDDT score of a decoy. We propose a novel loss function that captures our desiderata for a quality assessment model: when it comes to poor decoys, we do not care about the accuracy of the prediction as long as we can differentiate it from a good decoy. On the other hand, the more accurate the decoy, the more accurate we want our prediction to be. This is especially important given the recent improvement in the quality of protein structure prediction methods. To achieve this goal, we modify the *ϵ*-insensitive loss, which is the loss function employed in SVM regression (Drucker et al., 1996), as follows:

$$L\left(y,y^{\prime }\right)=\max\left(0,|y-y^{\prime }|-\epsilon \left(y\right)\right),$$ (3)

where *y* and *y*′ are the true and predicted scores, respectively. As in the standard *ϵ*-insensitive loss, this defines a tube of size *ϵ* within which there is no penalty; outside the tube, the loss grows linearly as in the L1-loss, which is defined as *L*(*y*, *y*′) = |*y* − *y*′|. In our application, the size of the tube is a function *ϵ*(*y*). In this work, we used a tube defined as shown in Figure 3. The motivation for the modified *ϵ*-insensitive loss function is that the model should not try too hard to accurately fit poor-quality decoys where we do not need good accuracy anyhow. As decoy quality increases, models are trained to learn a fit that is much more accurate.

### 3.6 Network training

We have trained our network to predict GDTTS and lDDT score. For GDTTS prediction, we first pre-train Q_*ϵ*_ with the L1-loss for 50 epochs, followed by training with the modified *ϵ*-insensitive loss for the next 10 epochs. To train the network with lDDT scores, we select the best model from GDTTS (“best” with respect to the validation set) and train it with the modified *ϵ*-insensitive loss for another 50 epochs, keeping the same network architecture and hyperparameters.

The network was implemented in PyTorch (Paszke et al., 2019) and optimized using the Adam method (Kingma and Ba, 2014) with default parameters except for a learning rate of 0.001; training used a batch size of 70. Since our training set is highly imbalanced, i.e., contains very few high-quality decoys, we used the imbalanced sampler from the torchsampler package. During training, we monitored the loss over the validation set and used the model that gave the minimum loss. Our implementation used the PyTorch Lightning framework for training and testing and PyTorch Geometric (Fey and Lenssen, 2019) for performing graph convolution. Model selection was performed using the hyperparameters and values described in Table 1. We iterated over all parameters and, for each one, chose the value that gave the highest Pearson correlation coefficient on the validation set. Following model selection, training took approximately 42 h on an NVIDIA RTX 3090 GPU.

**TABLE 1:** Hyperparameter space. Model selection was performed based on performance on the validation set.

Hyperparameter	Values	Best
Number of graph convolution layers	2, 3, 4, 5, 6	4
Neighbor distance threshold	4, 5, 6, 7, 8, 9	6
Maximum number of same residue atom neighbors	10, 15, 20, 25	20
Maximum number of different residue atom neighbors	10, 15, 20, 25	20
Maximum number of neighbors of a residue	10, 15, 20, 25	20
Dropout rate for the graph convolution layers	0, 0.1, 0.2, 0.3	0.1
Learning rate	0.0001, 0.001, 0.01, 0.1	0.001

The “Best” column provides the chosen value for each hyperparameter.

### 3.7 Data

We collected decoys from CASP9 to CASP14 along with their labels from the CASP website (CASP, 2021). We used CASP9–CASP12 as our training and validation sets and CASP13 and CASP14 as our test sets (see Table 2). In order to match the decoys used in experiments performed by others, we created two separate datasets for GDDTS evaluation (CASP13 and CASP14) and two datasets for the evaluation of lDDT score prediction (CASP13 and CASP14).

**TABLE 2:** Number of targets from CASP competitions in the training, validation, and testing data.

Mode	CASP	Target	Decoy
Training data	CASP9	117	31,863
	CASP10	100	23,755
	CASP11	84	15,573
	CASP12	30	5,351
Validation data	CASP12	10	1,338
Testing data (GDTTS)	CASP13	72	34,654
	CASP14	65	38,293
Testing data (lDDT score)	CASP13	76	10,739
	CASP14	70	10,380
	AlphaFold2 CASP15	17	85

Two different CASP13 and CASP14 datasets, one for GDTTS evaluation and the other for lDDT score evaluation, are used to match decoys used in other publications.

In CASP15, the focus shifted from predicting the accuracy of single-chain decoys to that of multi-chain complexes (Kryshtafovych et al., 2023). However, some of the targets were composed of single chains, and we chose to focus on those targets in our evaluation, leading to a dataset with 17 targets.

## 4 Results

We compare Q_*ϵ*_ with other methods that have either state-of-the-art or very good performance in CASP13 and CASP14. In our first set of experiments, we sought to compare our method with GraphQA, which uses a similar graph convolution architecture and was trained to predict GDTTS (Baldassarre et al., 2020). The results in Table 3 indicate that Q_*ϵ*_ outperforms GraphQA and several other recent methods trained to predict GDTTS despite not using engineered features; a detailed analysis of the contribution of the various components of the Q_*ϵ*_ architecture is described in an ablation study in the following section.

**TABLE 3:** Performance of Q_*ϵ*_ and other methods in CASP13 and CASP14 GDTTS prediction.

Dataset	Method	*R*	*R*_*target*_	*ρ*	RMSE
CASP13	Q_*ϵ*_	**0.90**	**0.80**	**0.89**	**0.10**
	GraphQA (Baldassarre et al., 2020)	0.86	0.78	0.86	0.13
	ModFOLD7_rank (McGuffin et al., 2019)	0.87	0.74	-	0.16
	ProQ4 (Hurtado et al., 2018)	0.70	0.66	-	0.18
	VoroMQA-A (Olechnovič and Venclovas, 2017)	0.66	0.56	-	0.21
CASP14	Q_*ϵ*_	**0.81**	**0.72**	**0.82**	**0.13**

The global Pearson correlation coefficient (*R*), Pearson correlation coefficient per target (*R*_target_), and Spearman rank correlation between predicted and known GDTTS are provided. The best performance is highlighted in bold. Performance numbers for the other methods is quoted from Baldassarre et al. (2020).

The quality assessment community is transitioning to the use of the lDDT score, so we also compare Q_*ϵ*_ with more recent methods evaluated with lDDT. In this evaluation, the performance of Q_*ϵ*_ was similar to that of DeepUMQA but outperformed by its successor, DeepUMQA2 (see Table 4). Results from EnQA (Chen et al., 2023), whose performance was similar to that of DeepUMQA2, are also better than those of Q_*ϵ*_. Both methods use more complex architectures and extensive engineered features; DeepUMQA2 also used evolutionary information, including structural features from homologous templates.

**TABLE 4:** Performance of Q_*ϵ*_ and other methods in CASP13 and CASP14 with respect to lDDT scores.

Dataset	Method	*R*	*ρ*
CASP13	Q_*ϵ*_	0.857	**0.862**
	DeepUMQA2 (Liu et al., 2023)	**0.919**	-
	DeepUMQA (Guo et al., 2022)	0.837	0.804
	ModFOLD7_rank (Maghrabi and McGuffin, 2020)	0.826	-
	ProQ3D (Uziela et al., 2017)	0.801	-
	ProQ4 (Uziela et al., 2017)	0.777	-
	ProQ2 (Ray et al., 2012)	0.715	-
	VoroMQA-A (Olechnovič and Venclovas, 2017)	0.672	-
CASP14	Q_*ϵ*_	0.826	**0.826**
	DeepUMQA2 (Liu et al., 2023)	**0.899**	-
	DeepUMQA (Guo et al., 2022)	0.799	0.736
	DeepAccNet (Hiranuma et al., 2021)	0.829	-
	ModFOLD8 (McGuffin et al., 2021)	0.629	-
	GraphQA (Baldassarre et al., 2020)	0.706	-
	ProQ3D (Uziela et al., 2017)	0.717	-
	ProQ2 (Ray et al., 2012)	0.531	-
	ProQ4 (Uziela et al., 2017)	0.547	-

The global Pearson correlation coefficient (*R*) and Spearman rank correlation between predicted and known lDDT scores are provided. The best performance is highlighted in bold. Performance figures for methods other than Q_*ϵ*_ are quoted from Guo et al. (2022) and Liu et al. (2023).

**FIGURE 4:**
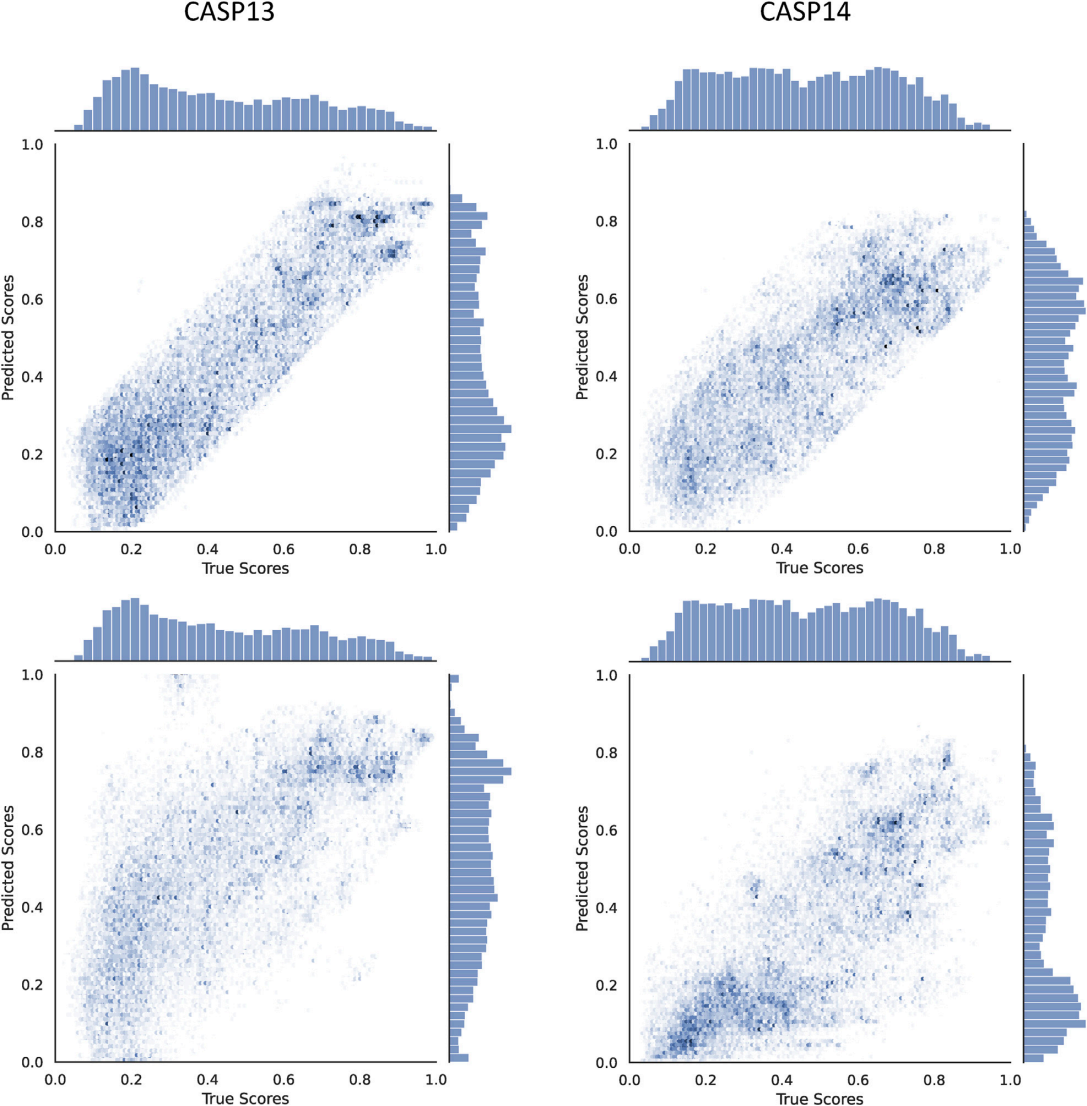
Scatter plots comparing the true and predicted GDTTS for both CASP13 and CASP14 using L1-loss (bottom) and modified ε-insensitive loss (top).

To understand the contribution of the proposed modified *ϵ*-insensitive loss to the performance of Q_*ϵ*_, a scatter plot of true versus predicted GDTTS for the decoys in CASP13 and CASP14 is shown in Figure 4. We observe that the modified *ϵ*-insensitive loss leads to better learning of decoys of all quality levels compared to the L1-loss and leads to a pattern where the predictions are limited to a band around the true scores, which is a highly desirable property for a quality assessment method. It was interesting that the width of the band is similar across all quality levels, despite the loss having a variable width band compared to the original *ϵ*-insensitive loss function.

### 4.1 Ablation study

To demonstrate the contribution of each of the major components of our method, we performed an ablation study with respect to GDTTS prediction, and its results are shown in Table 5. The first component we varied was the loss function. We observe that the pre-training with the L1-loss is key for the method’s performance, serving to bootstrap the learning process. We also observe that performance dropped when using the original *ϵ*-insensitive loss function, L1-loss, or L2-loss. This clearly shows the contribution of the proposed modification to the *ϵ*-insensitive loss. Our next observation is that both the residue-level and atom-level convolutional blocks are crucial for the performance of the method. This is due to each of them providing different and complementary information. The residue-level blocks use ProtTrans embeddings, which have been documented to provide a variety of information regarding a residue’s evolutionary history and structural context within the protein (Elnaggar et al., 2021). The atom-level convolutional blocks provide a more fine-grained view of a decoy structure, complementing the information at the residue level.

**TABLE 5:** Q_*ϵ*_ ablation study.

Method	*R*	*R*_*target*_	*ρ*	RMSE
Q_*ϵ*_ (with atom and residue features, pre-trained with L1-loss and modified *ϵ*-insensitive loss)	**0.90**	**0.80**	**0.89**	**0.11**
Q_*ϵ*_ without modified *ϵ*-insensitive loss	0.75	0.66	0.69	0.17
Q_*ϵ*_ without L1-loss	0.70	0.59	0.62	0.20
Q_*ϵ*_ with a constant *ϵ* (0.2)	0.63	0.55	0.66	0.24
Q_*ϵ*_ with only L2-loss	0.65	0.52	0.56	0.23
Q_*ϵ*_ without residue features	0.70	0.65	0.69	0.19
Q_*ϵ*_ without atom features	0.79	0.77	0.76	0.18

Each of the major elements of Q_*ϵ*_ is removed, demonstrating that each of them provides a major contribution to the performance of the method.

### 4.2 *ϵ*-threshold selection

The modified *ϵ*-insensitive loss has nine threshold parameters associated with the epsilon insensitive loss function, one for each bin of the prediction score. In our experiments, we have used the values shown in Figure 3. In order to determine that our initial choice was good, we ran an experiment where we varied all the values in a coordinated manner: we chose nine values lower or higher than the initial values (the columns low and high in Table 6). As shown in Table 6, reducing or increasing the values of all the thresholds in a coordinated fashion led to reduced accuracy on the validation set. As a sanity check, we verified that a similar decrease is observed on the test set as well.

**TABLE 6:** Model selection over the *ϵ* hyperparameter values.

Score ranges and results	Low value	Mid value	High value
*ϵ* for 0–0.1	0.40	0.45	0.50
*ϵ* for 0.1–0.2	0.35	0.40	0.45
*ϵ* for 0.2–0.3	0.30	0.35	0.40
*ϵ* for 0.3–0.4	0.25	0.30	0.35
*ϵ* for 0.4–0.5	0.20	0.25	0.30
*ϵ* for 0.5–0.6	0.15	0.2	0.25
*ϵ* for 0.6–0.7	0.10	0.15	0.20
*ϵ* for 0.7–0.8	0.05	0.1	0.15
*ϵ* for > 0.8	0.005	0.01	0.015
R on CASP12 (validation set) (GDTTS)	0.84	0.89	0.82
R on CASP12 (validation set) (lDDT score)	0.81	0.84	0.77
R on CASP13 (test set) (GDTTS)	0.86	0.90	0.85
R on CASP14 (test set) (GDTTS)	0.80	0.81	0.79
R on CASP13 (test set) (lDDT score)	0.84	0.86	0.83
R on CASP14 (test set) (lDDT score)	0.82	0.83	0.80

The top half shows the values of *ϵ* for each score range. The lower half shows the performance for each combination of values (low/mid/high); R stands for the Pearson correlation coefficient. Results are shown for the validation set (first two rows) and the test set for both GDTTS and lDDT score.

### 4.3 Local quality assessment with Q_*ϵ*_

In this section, we demonstrate the ability of Q_*ϵ*_ to make accurate predictions at the residue level, despite being trained only on global quality scores. This ability is a byproduct of the architecture of the network, where the global predicted score is an average of residue-level node summary scores (see Figure 2). This forces the network to learn accurate local scores, as demonstrated in the results shown in Table 7. Similar to the global prediction problem, the performance of Q_*ϵ*_ is between that of DeepUMQA and DeepUMQA2.

**TABLE 7:** Performance of Q_*ϵ*_ and other methods in CASP13 and CASP14 with respect to local lDDT scores.

Dataset	Method	*R*_*local*_
CASP13	*Q*_*ϵ*_	0.80
	DeepUMQA2 (Liu et al., 2022)	**0.868**
	DeepUMQA (Guo et al., 2022)	0.766
	DeepAccNet (Hiranuma et al., 2021)	0.740
CASP14	*Q*_*ϵ*_	0.76
	DeepUMQA2 (Liu et al., 2022)	**0.822**
	DeepUMQA (Guo et al., 2022)	0.680
	DeepAccNet (Hiranuma et al., 2021)	0.672

The local Pearson correlation coefficient (*R*_*local*_) between predicted and known local lDDT scores is provided. The best performance is highlighted in bold. Performance figures for methods other than Q_*ϵ*_ are quoted from Liu et al. (2022).

### 4.4 Results on CAMEO decoys

For further validation of the performance of Q_*ϵ*_, we evaluated its performance on decoys from the CAMEO evaluation project (Haas et al., 2018). We downloaded decoys used from 13 May 2022 to 06 May 2023 and followed the same evaluation protocol used by CAMEO: the area under the ROC (AUROC) curve and area under the precision recall (AUPR) curve were calculated using a local lDDT score threshold of 0.6, and the obtained results are shown in Table 8. Again, we note that Q_*ϵ*_ was not trained on local scores (unlike the other methods) and yet is able to perform almost on par with DeepUMQA2. As mentioned previously, this can be traced to the fact that the global prediction score computed by Q_*ϵ*_ is evaluated by directly averaging local node summary scores, forcing those scores to reflect a local measure of quality.

**TABLE 8:** Performance of Q_*ϵ*_ and other methods on the CAMEO dataset.

Dataset	Method	Model	AUROC	AUPR
CAMEO-QA	Q_*ϵ*_	6,350	0.93	0.88
	DeepUMQA2 (Liu et al., 2023)	6,225	**0.94**	**0.89**
	ProQ3D_LDDT (Uziela et al., 2017)	6,498	0.90	0.81
	DeepUMQA (Guo et al., 2022)	6,247	0.93	0.86
	ModFOLD9 (McGuffin et al., 2023)	6,498	0.92	0.87

The best performance is highlighted in bold. All other results have been taken from the CAMEO website.

### 4.5 Performance on AlphaFold2 decoys

In CASP14, AlphaFold2 provided, for the first time, decoys with near experimental resolution (Skolnick et al., 2021), with a median GDTTS of 92.4, making it the first team to achieve the highest level of accuracy in CASP. We gathered the decoys submitted by the AlphaFold team (team no 427) from the CASP14 website and evaluated Q_*ϵ*_ on their decoys. We also ran AlphaFold2 version 2.3.1 on CASP15 single-chain targets. The results of this experiment are shown in Table 9. While AlphaFold2 provided better accuracy than our method, its value provided independent validation for the quality of AlphaFold2 predictions. EnQA (Chen et al., 2023) slightly improves on the quality of AlphaFold2 lDDT score estimates; however, it does so by using the AlphaFold2 scores as one of its features. Therefore, the results of the EnQA method are expected to be highly correlated with those of AlphaFold2 and less useful for independent verification of its predictions.

**TABLE 9:** Performance of Q_*ϵ*_ and AlphaFold2 on AlphaFold2-generated decoys in CASP14 and CASP15.

Dataset	Method	*R*	*R*_*local*_	*ρ*
AlphaFold2-CASP14	*Q*_*ϵ*_	0.772	0.730	0.832
	AlphaFold2	**0.85**	**0.792**	**0.882**
AlphaFold2-CASP15	*Q*_*ϵ*_	0.64	0.60	0.60
	AlphaFold2	**0.75**	**0.72**	**0.67**

The global Pearson correlation (*R*), local Pearson correlation (*R*_*local*_), and Spearman rank correlation (*ρ*) between predicted and known local and global lDDT scores are provided. The best performance is highlighted in bold.

## 5 Conclusion and future work

In this study, we proposed a novel loss function to enhance the performance of deep learning for quality assessment of decoy structures. Our approach performed close to other state-of-the-art methods while at the same time removing the need for engineered features computed from the protein structure, relying solely on features computed from the decoy sequence, demonstrating what is possible with a pure deep learning approach. These features were integrated using graph convolutional layers that operate at both the atom and residue levels, thereby improving the network’s performance. The comparison of our approach with AlphaFold2 indicates there is a need for further research to provide accuracy estimates that improve on the local scores computed by AlphaFold2 in order to provide independent validation of the quality of its predicted structures.

Our approach can be extended in multiple ways. First, although it performs well in predicting local scores, the method is trained using only global quality scores. Joint learning of both global and local scores can potentially improve performance for both tasks. Second, we treated the prediction of GDDTS and lDDT score as independent tasks; there is a potential gain in addressing multiple quality scores at the same time (Baldassarre et al., 2020). Finally, in this work, we chose to focus on the contribution of the loss function to method performance, so we used a relatively simple graph convolutional network similar to that used in GraphQA (Baldassarre et al., 2020). Finally, we expect that the proposed loss function can be applied to regression problems, whose objective is to detect high-quality objects, and has the potential to be a useful addition to any deep learning toolbox.

## Data Availability

The datasets presented in this study can be found in online repositories. The names of the repository/repositories and accession number(s) can be found in the article/Supplementary Material.
